# Global miRNA, lncRNA, and mRNA Transcriptome Profiling of Endometrial Epithelial Cells Reveals Genes Related to Porcine Reproductive Failure Caused by Porcine Reproductive and Respiratory Syndrome Virus

**DOI:** 10.3389/fimmu.2019.01221

**Published:** 2019-06-04

**Authors:** Kang Zhang, Lijiang Ge, Shasha Dong, Ying Liu, Dong Wang, Chunyan Zhou, Cai Ma, Yanchao Wang, Feng Su, Yunliang Jiang

**Affiliations:** ^1^Shandong Provincial Key Laboratory of Animal Biotechnology and Disease Control and Prevention, College of Animal Science and Technology, Shandong Agricultural University, Taian, China; ^2^Department of Cardiology, Shandong First Medical University and Shandong Academy of Medical Science, Taian, China

**Keywords:** PRRSV, PECs, lncRNA, mRNA, integrated analysis

## Abstract

Porcine reproductive and respiratory syndrome virus (PRRSV) can cause respiratory disease and reproductive failure in pregnant pigs. Previous transcriptome analyses in susceptive cells have mainly concentrated on pulmonary alveolar macrophages (PAM) and Marc-145 cells, and on the respiratory system. Some studies reported that apoptosis of placental cells and pig endometrial epithelial cells (PECs) is an obvious sign linked to reproductive failure in pregnant sows, but the mechanism is still unknown. In this study, Sn-positive PECs were isolated and apoptosis rates were assessed by flow cytometry. PRRSV-infected PECs exhibited apoptosis, indicative of their susceptibility to PRRSV. Subsequently, the whole transcriptome was compared between mock- and PRRSV-infected PECs and 54 differentially expressed microRNAs (DEmiRNAs), 104 differentially expressed genes (DEGs), 22 differentially expressed lncRNAs (DElncRNAs), and 109 isoforms were obtained, which were mainly enriched in apoptosis, necroptosis, and p53 signal pathways. Integration analysis of DEmiRNA and DEG profiles revealed two microRNAs (*ssc-miR-339-5p* and *ssc-miR-181d-5p*) and five genes (*SLA-DQB1, THBS1, SLC3A1, ZFP37*, and *LOC100517161*) participating in the apoptosis signal, of which *THBS1* and *SLC3A1* were mainly linked to the p53 pathway. Integration analysis of DEGs with DElncRNA profiles identified genes involved in apoptosis signal pathway are regulated by *LTCONS_00010766* and *LTCONS_00045988*. Pathway enrichment revealed that the phagosome and p53 pathways are the two main signals causing apoptosis of PECs, and functional analysis revealed a role of *miR-339-5p* in regulating apoptosis of PECs after PRRSV inoculation.

## Introduction

Porcine reproductive and respiratory syndrome (PRRS), an infectious viral disease, results in tremendous economic loss in the swine industry, through reproductive failure in breeding sows, and respiratory disorders in young and growing pigs (1). The porcine reproductive and respiratory syndrome virus (PRRSV) mainly causes reproductive failure in pregnant sows in the late period (2). Previous studies have reported that PRRSV exhibits infection permissiveness causing CD163^+^ and Sn^+^ lymphocyte apoptosis (3). The distribution of these positive cells explains the infectious attributes of PRRSV, which ultimately causes reproductive failure (3). Subsequently, in assays carried out by Feng and colleagues, CD163^+^ pig endometrial epithelial cells (PECs) were isolated and it was verified that PRRSV could replicate in these cells (4). A recent study showed that PRRSV could cause placental cell and PEC apoptosis and autophagy in the implantation site, leading to reproductive failure in the later phases of pregnancy (5).

Placental cell and PEC apoptosis have been recognized as obvious signs linked to reproductive failure in pregnant sows (6, 7). In a previous study, it was reported that PRRSV could cause apoptosis of Marc-145 cells through a mitochondria-mediated pathway (8). More recently, PRRSV was confirmed to lead not only to apoptosis, but also to autophagy of Marc-145 cells; in addition, cell apoptosis was proven to be the major reason for reduction of virus replication (9). In a study by Huo and colleagues, p53 protein was activated in PRRSV-infected Marc-145 cells and the activation of the JNK pathway induced cell apoptosis (10, 11). However, these studies mainly focused on Marc-145 cells and PAM cells, and no assays were reported to explain the mechanism underlying PRRSV-induced PEC apoptosis.

Cell apoptosis is a complicated process that is regulated by multiple factors. Expression of mRNA and miRNA in target cells in response to PRRSV has been investigated and differentially expressed mRNAs and differentially expressed miRNAs (DEmiRNAs) have been screened (12–17). However, the identified mRNAs and miRNAs were mainly related to PRRSV replication and organism immunity, and few were related to apoptosis caused by PRRSV. The lncRNA profile changes of PRRSV-infected PAM cells were investigated and a series of lncRNAs were screened and identified (12). Consistent with miRNA and mRNA studies, the identified lncRNAs were mainly involved in immune system. Some miRNAs were found to be involved in apoptosis and act as regulators of the mitochondrial apoptosis pathway (18–20). Some lncRNAs have been reported as negative regulators and taking part in the cell apoptosis pathway (20–22). However, the DEmiRNAs and differentially expressed lncRNAs (DElncRNAs) that participate in PRRSV-infected PECs are still unknown.

In this study, we isolated Sn^+^ PECs and reported what is believed to be the first comprehensive and integrative analysis of the mRNAs, miRNAs, and lncRNAs underlying apoptosis in PRRSV-infected PECs. Changes in some mRNA and miRNA relating to the key genes of PRRSV-infected PECs were also characterized. Moreover, using these data, we have unveiled several candidate genes and signaling pathways related to apoptosis of PECs caused by PRRSV.

## Materials and Methods

### Ethics Statement

All sows used in this study were housed in livestock housing and fed *ad libitum*. The sacrifice of sows was carried out with sodium barbital after anesthesia. All procedures involving animals were approved by the Animal Care and Use Committee of Shandong Agricultural University.

### Cell Culture and Isolation

Sows of the Large White pig breed were used, which had not been vaccinated against PRRSV since birth. The sows were sacrificed at the age of 4 months. The uterus of each pig was removed and used for cell culture. The endometrium epithelial layer was isolated and cut into 1 mm^3^ cubes, then the cells were cultured in DMEM-F12 medium containing 10% fetal bovine serum (Gibco, Invitrogen, Carlsbad, CA, USA) and epidermal growth factor (10 ng/mL; Sigma, USA) using the tissue explant adherence method. After PEC clones had formed and expanded, Sn protein was used as a specificity marker for PEC identification. Briefly, the cells were fixed with polyformaldehyde for 24 h, and then the cells were incubated with Cytokeratin-18 (Beyotime, Jiangsu, China) and Sialoadhesin antibodies (ab94715; ABcom, Cambridge, USA) separately, after being incubated with blocking buffer (Beyotime, Jiangsu, China) for 4 h. The cells were then incubated with goat anti-rabbit antibody for 2 h, after being washed with washing buffer three times. The cells were then examined microscopically.

### PRRSV Infection and Cell Apoptosis Analysis

PRRSV was kindly donated by Dr. Xiao of Shandong Agricultural University. PRRSV infection and titration were performed as described previously (23). Rates of PRRSV-induced cell apoptosis in PECs were determined by flow cytometry using the Annexin V-FITC Apoptosis Detection Kit (Beyotime), following the manufacturer's instructions. Briefly, PECs were incubated with PRRSV for 24 h, washed twice with ice-cold PBS, and then 5 μL of annexin V-FITC and 1 μL of PI (1 mg/mL) were applied to stain the cells. The stained cells were analyzed using a flow cytometer.

### Sample Collection and Preparation

Isolated porine endometrial cells were divided into two groups. One group was defined as the control group (without PRRSV infection) and the other as the experiment group (infected with PRRSV, as mentioned above). All the samples were sent to Beijing Genomics Institute (BGI) for entire transcriptome sequencing. Each group consists of three technical replicate samples.

### Total RNA Extraction

Total RNA was extracted using TRIzol reagent (Sigma) and treated with DNase to remove potential genomic DNA contamination, following the manufacturer's protocol. The quantity and purity of the total RNA were evaluated. RNA integrity was checked by microcapillary electrophoresis using an Agilent 2100 Bioanalyzer with an RNA 6000 Nanochip kit. The RNA was then divided into two aliquots that were used for library construction of either small RNA or RNA.

### Preparation of the RNA-Seq Library

Total RNA was divided into two samples after preparation. In one sample, ribosomal RNA was removed by Epicenter Ribo-zero™ rRNA Removal Kit (Epicenter, Madison, WI, USA), and residual RNAs were cleaned by ethanol precipitation. The sequencing libraries were generated using rRNA-depleted RNA with a NEB Next® Ultra™ Directional RNA Library Prep Kit for Illumina® (NEB, USA). The constructed libraries were evaluated on an Agilent Bioanalyzer 2100 system (12). RNA integrity was checked by microcapillary electrophoresis using an Agilent 2100 Bioanalyzer with an RNA 6000 Nanochip kit (Agilent Technologies, Germany) (15). Sequencing was then performed using a paired-end 125-cycle rapid run on an Illumina HiSeq2500 (Illumina Inc., San Diego, CA, USA). Low-quality reads were removed, and the clean reads were filtered from the raw reads and mapped to the porcine reference genome (Sus scrofa v10.2). The mapped reads for each sample were independently assembled using Cufflinks (v2.1.1).

### RNA-seq Data Analysis

The raw sequencing data (raw reads) were preserved in FASTQ format. Clean data of high quality were then aligned to the *Sus scrofa* genome assembly (Sus Scrofa v10.2) using TopHat2 (v2.0.9) (24). The transcriptome of each sample was assembled from the mapped reads using Cufflinks (v2.1.1) (25).

### Initial Screening of miRNAs and RNAs

Significantly differentially regulated miRNAs and RNAs were screened in several steps. Firstly, the miRNAs and RNAs with no signals to the background were excluded in each group. Secondly, the differentially expressed miRNAs and RNAs were screened by a parametric t-test with a Benjamini–Hochberg adjusted significance level of 0.001. A usual selection criterion for biomarkers was set at an alpha level of 0.05 for Benjamini–Hochberg adjusted significance values. The relative expression levels of miRNAs were then normalized as the trimmed mean of M-values (TMM) using the edge R package. An absolute value of log2 FC ≥ 2 and an FDR < 0.01 was considered as significantly differentially expressed compared with the control group.

### Gene Expression Analysis

The transcriptome data have been deposited in the National Center for Biotechnology Information Gene Expression Omnibus (GEO, https://www.ncbi.nlm.nih.gov/sra) under the accession number SRP158168. Gene expression levels were estimated by fragments per kilobase per million (FPKM) values obtained using Cufflinks software. The discrepant genes were analyzed only with an absolute value of log2 FC ≥ 2 and an FDR < 0.01.

### Prediction of the Function of lncRNAs

Prediction of the functions of lncRNAs was performed using their related *cis*- and *trans*-target mRNAs that were functionally well-annotated. Potentially cis-regulated target genes were deemed as 10 kb in genomic distance from the lncRNA and potentially trans-regulated target genes were identified using RNAplex software (26, 27).

### GO (Gene Ontology) and KEGG Enrichment Analysis

DElncRNA, DEmiRNA, and DEGs were screened. GO and KEGG analyses of the differentially expressed genes (DEGs) were carried out with the GO seq R package (v1.18.0) (28) and KOBAS software (v2.0) (29).

### Integrated Analysis of DEGs and DElncRNA Target Genes

Based on the competing endogenous RNA (ceRNA) hypothesis, we constructed DEGs and DElncRNAs crosstalk networks. The networks were constructed by integrating prior knowledge of miRNA and lncRNA interactions (30).

### Real-Time PCR Analysis

Real-time PCR was performed using SYBR® Green PCR Master Mix (TaKaRa, Dalian, China) and an Applied Biosystems 7500 Real-Time PCR System. All primers used in this study are listed in Table 1.

**Table 1:** Primers used in this study.

**Genes**	**Strand**	**Sequence (5^**′**^-3^**′**^)**	**Length**	**Annealing temperature** **(^**°**^C)**
Loc100517161	F	AGGCTCACTGTCATTCAAG	290	60
	R	CCATAGTTTCCAGGTTGC		
SLC3A1	F	GTGGCTTCTGTGCTTGCG	140	60
	R	CCGTTCCCGTCTTTGTCG		
THBS1	F	CAATCCTTGCTTTGCTGGTG	264	59
	R	TTGTTGGCGGTGGCGTAT		
SLA-DQB1	F	CAGATAGAGGAAGGCACGACC	138	61
	R	GACTTTCACCTGGCTTGGATAG		
ZFP37	F	TGAGAAGTTATCCAACCGTAGC	391	60
	R	ATGGTCAGTGAGGGCGTGT		
GAPDH	F	TGGTGAAGGTCGGAGTGAAC	225	60
	R	GGAAGATGGTGATGGGATTTC		
ssc-miR-181d-5p	F	CATTCATTGTTGTCGGTGGGTT		60
ssc-miR-339-5p	F	GATTCCAGGAGCTCACGAAA		60
U6	F	CTCGCTTCGGCAGCACA		60
	R	AACGCTTCACGAATTTGCGT		
miR-339-5p inhibitor		GUGAGCUCCUGGAGGACAGGGA		

### RNA Interference and Western Blotting

The small interfering RNA to reduce the expression of *miR-339-5p* was synthesized (Table 1) and transfected into PECs. After PECs were infected PRRSV, apoptosis were analyzed as described above. Subsequently, the Caspase 3 and Caspase 8 protein were detected by Western blotting. After extracted protein by PIPA (Beyotime, Jiang Su,China), the protein were then checked by SDS-PAGE. After the protein were transferred into PVDF membrane. The Caspase 3 antibody (Cat: ab2302, Abcam) and Caspase 8 antibody (Cat: ab25901, Abcam) were incubated with the membrane separately. The membrane were exposed after incubated with Goat anti-rabbit antibody (Cat: ab6721,Abcam).

### Statistical Analysis

DEG expression analysis results are presented as the mean ± SEM and were analyzed by one-way ANOVA test. GO and KEGG analyses were assessed by Fisher's *t*-test. A *P* < 0.05 was considered as significantly different. All of the co-expressed relationships were predicted using Cytoscape ClueGO plug-in (v2.3.2, http://apps.cytoscape.org/apps/cluego) as a complementary analysis method. Only Benjamini–Hochberg-corrected values of *P* < 0.05 were considered statistically significant.

## Results

### Isolation and Purification of PRRSV-Susceptive PECs

**Figure 1:**
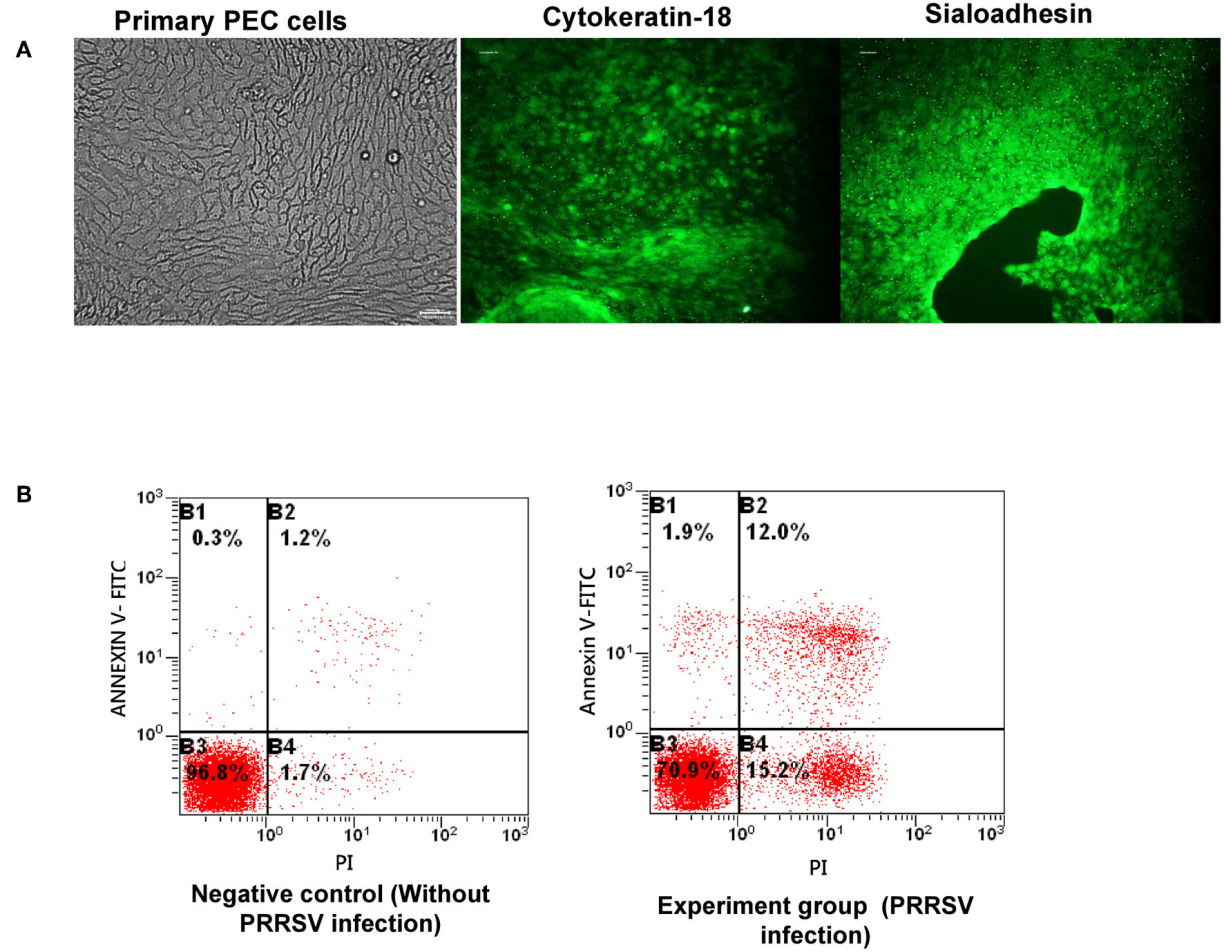
Isolation and purification of PECs and infection capacity evaluation. **(A)** Isolation and verification of PECs by immunofluorescence. The left image represents isolated PECs from the uterus. The middle image illustrates expression of the cytokeratin-18 marker in PECs. The right image illustrates expression of the sialoadhesin marker in PECs. **(B)** Apoptosis ratio analysis of PECs infected with PRRSV (MOI 10:1). The left image shows PECs without PRRSV infection. The right image shows PECs infected with PRRSV (MOI 10:1).

PECs were isolated, purified, and then dyed with Keratin-18 and Sialoadhesin antibodies, separately. The green fluorescence shown in Figure 1A indicated that the isolated cells were Keratin-18 and Sialoadhesin positive, suggesting a potential PRRSV infectivity of them. Subsequently, the PRRSV susceptibility of PECs was evaluated by assessing the apoptosis of PRRSV infected PECs. The rate of apoptosis caused by PRRSV was obviously increased, indicating that the cells isolated were susceptible to PRRSV (Figure 1B).

### Landscape of the miRNA Transcriptomes in PECs

**Figure 2:**
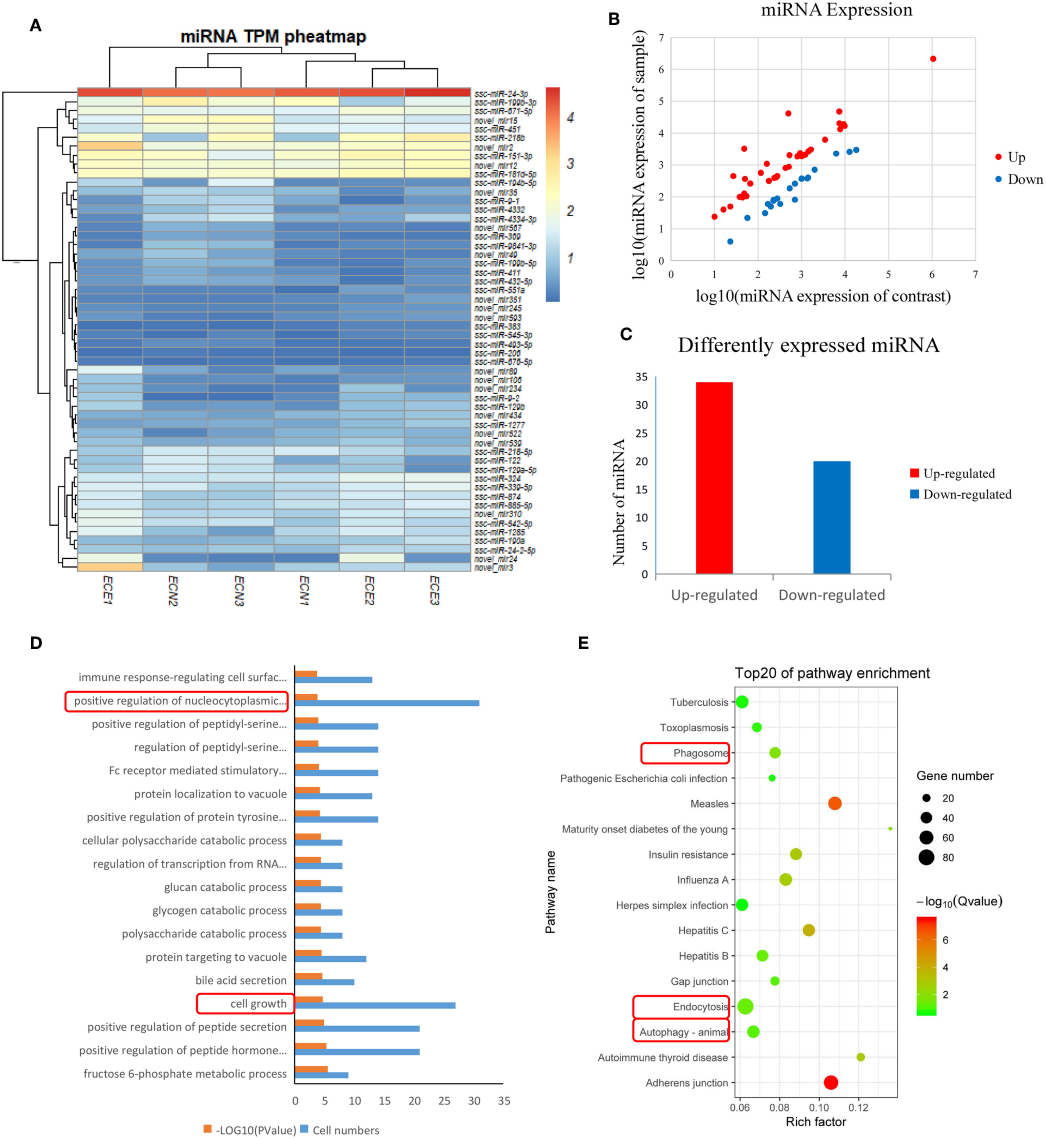
Screening and enrichment analysis of differently expressed miRNA (DEmiRNA) in PRRSV-infected PECs compared with non-infected PECs. **(A)** Hierarchical clustering analysis (heatmap) for DEmiRNAs using Pearson's correlation. **(B)** DEmiRNA expression level analysis by scatter plot. The red spots in this figure represent up-regulated miRNAs, the blue spots represent down-regulated miRNAs. **(C)** Detailed analysis on the up-/down-regulated DEmiRNAs. Thirty-four up-regulated miRNAs and 20 down-regulated miRNAs were shown. **(D)** The first 20 GO terms analysis (*P* < 0.005) of DEmiRNAs in biological processes. The red textbox in the figure indicates the main processes that related into cells grow and apoptosis. A –log_10_ (*P*-value) > 2 was considered as significantly different. **(E)** KEGG signal pathway enrichment analysis of DEmiRNAs (*P* < 0.01). The pathways labeled with red textbox were important pathways that are related to cells apoptosis.

We generated six miRNA expression profiles of PECs from Large White pigs, three of each from mock- and PRRSV-infected PECs, respectively. Clean reads were obtained after filtering for reads with low quality and removing adaptor sequences from the raw reads (Supplemental Table 1). A total of 54 DEmiRNAs were obtained (Table 2) and a heatmap is shown in Figure 2A. miRNA expression levels are illustrated by scatter plot in Figure 2B. Detailed analysis of the up-/down-regulated DEmiRNAs is shown in Figure 2C. The observed up-regulation of specific DEmiRNAs might contribute to reproductive failure in pigs caused by PRRSV infection.

**Table 2:** Differential miRNA expression.

**miRNA id**	**log2Ratio** **(Sample/control)**	**Up/down regulation**	***P*-value**	***Q*-value**
novel_mir15	−1.944690794	DOWN	0	0
novel_mir35	−1.085608983	DOWN	2.51E-28	2.59E-28
novel_mir49	−1.29065831	DOWN	1.79E-15	1.58E-15
novel_mir567	−1.62350323	DOWN	1.93E-15	1.70E-15
ssc-miR-122	−1.488998972	DOWN	8.98E-86	1.30E-85
ssc-miR-129a-5p	−1.454395851	DOWN	3.62E-86	5.27E-86
ssc-miR-194b-5p	−1.057208557	DOWN	3.00E-38	3.51E-38
ssc-miR-199b-3p	−2.255474013	DOWN	0	0
ssc-miR-199b-5p	−1.218503253	DOWN	7.17E-12	5.52E-12
ssc-miR-206	−2.176211531	DOWN	0.000915313	0.000341273
ssc-miR-218-5p	−1.142424055	DOWN	1.04E-83	1.48E-83
ssc-miR-369	−1.878362355	DOWN	3.86E-14	3.26E-14
ssc-miR-411	−1.138076402	DOWN	2.03E-08	1.32E-08
ssc-miR-432-5p	−1.145689587	DOWN	5.04E-11	3.71E-11
ssc-miR-4332	−1.174186696	DOWN	1.14E-24	1.14E-24
ssc-miR-4334-3p	−1.07982083	DOWN	4.06E-39	4.78E-39
ssc-miR-451	−1.098656926	DOWN	8.88E-242	2.09E-241
ssc-miR-493-5p	−1.026107971	DOWN	0.002667718	0.000908341
ssc-miR-9-1	−2.746308682	DOWN	4.32E-97	6.54E-97
ssc-miR-9841-3p	−2.081149334	DOWN	1.37E-33	1.52E-33
novel_mir106	2.336379307	UP	1.16E-39	1.37E-39
novel_mir12	1.139769481	UP	0	0
novel_mir2	3.044520211	UP	0	0
novel_mir234	3.219194073	UP	9.44E-76	1.31E-75
novel_mir24	6.425946028	UP	0	0
novel_mir245	1.30670844	UP	2.44E-08	1.58E-08
novel_mir3	6.731975673	UP	0	0
novel_mir310	1.694605865	UP	3.33E-225	7.53E-225
novel_mir351	1.487831648	UP	3.31E-09	2.25E-09
novel_mir434	1.017042664	UP	1.54E-19	1.45E-19
novel_mir522	1.19262783	UP	1.62E-19	1.52E-19
novel_mir539	1.064241579	UP	8.39E-23	8.18E-23
novel_mir593	1.762387924	UP	1.65E-14	1.42E-14
novel_mir89	3.129864492	UP	5.39E-217	1.20E-216
ssc-miR-1277	1.096542321	UP	4.73E-21	4.56E-21
ssc-miR-1285	2.30004471	UP	1.34E-290	3.49E-290
ssc-miR-129b	2.639116549	UP	4.42E-96	6.63E-96
ssc-miR-151-3p	1.405595005	UP	0	0
ssc-miR-181d-5p	1.12718902	UP	0	0
ssc-miR-190a	1.269254058	UP	9.30E-52	1.19E-51
ssc-miR-218b	1.813324005	UP	0	0
ssc-miR-24-2-5p	1.140463424	UP	8.86E-48	1.11E-47
ssc-miR-24-3p	1.363494934	UP	0	0
ssc-miR-324	1.255654734	UP	1.61E-187	3.17E-187
ssc-miR-339-5p	1.243201213	UP	4.13E-162	7.73E-162
ssc-miR-383	1.66927852	UP	3.63E-05	1.72E-05
ssc-miR-542-5p	1.147380709	UP	6.74E-113	1.08E-112
ssc-miR-545-3p	1.467644658	UP	2.62E-05	1.26E-05
ssc-miR-551a	1.743279101	UP	1.66E-11	1.26E-11
ssc-miR-671-5p	1.198396549	UP	0	0
ssc-miR-676-5p	1.610384831	UP	0.001821424	0.000649716
ssc-miR-874	1.565878137	UP	1.11E-159	2.05E-159
ssc-miR-885-5p	1.250986748	UP	2.49E-115	4.07E-115
ssc-miR-9-2	4.409446546	UP	6.45E-115	1.04E-114

### Functional Annotation of the Target Genes of Specific DEmiRNAs

DEmiRNAs were predicted by the validated miRNA-targets database (mirTarBase 4.5) and 1,453 targets were captured (Supplemental Table 2). Subsequently, the target genes were analyzed by GO and KEGG analyses. Figure 2D shows the first 20 GO terms (*P* < 0.005) from a biological process analysis (Supplemental Table 3). The GO analysis showed that the target genes were significantly enriched in cell growth (*P* = 0.0000186) and positive regulation of nucleocytoplasmic transport (*P* = 0.00018). KEGG analysis of DEmiRNAs suggested that cytophagy (phagosome and endocytosis pathways) and autophagy were the main pathways influencing cell apoptosis (Figure 2E).

### Summary of RNA-Seq in PECs

**Figure 3:**
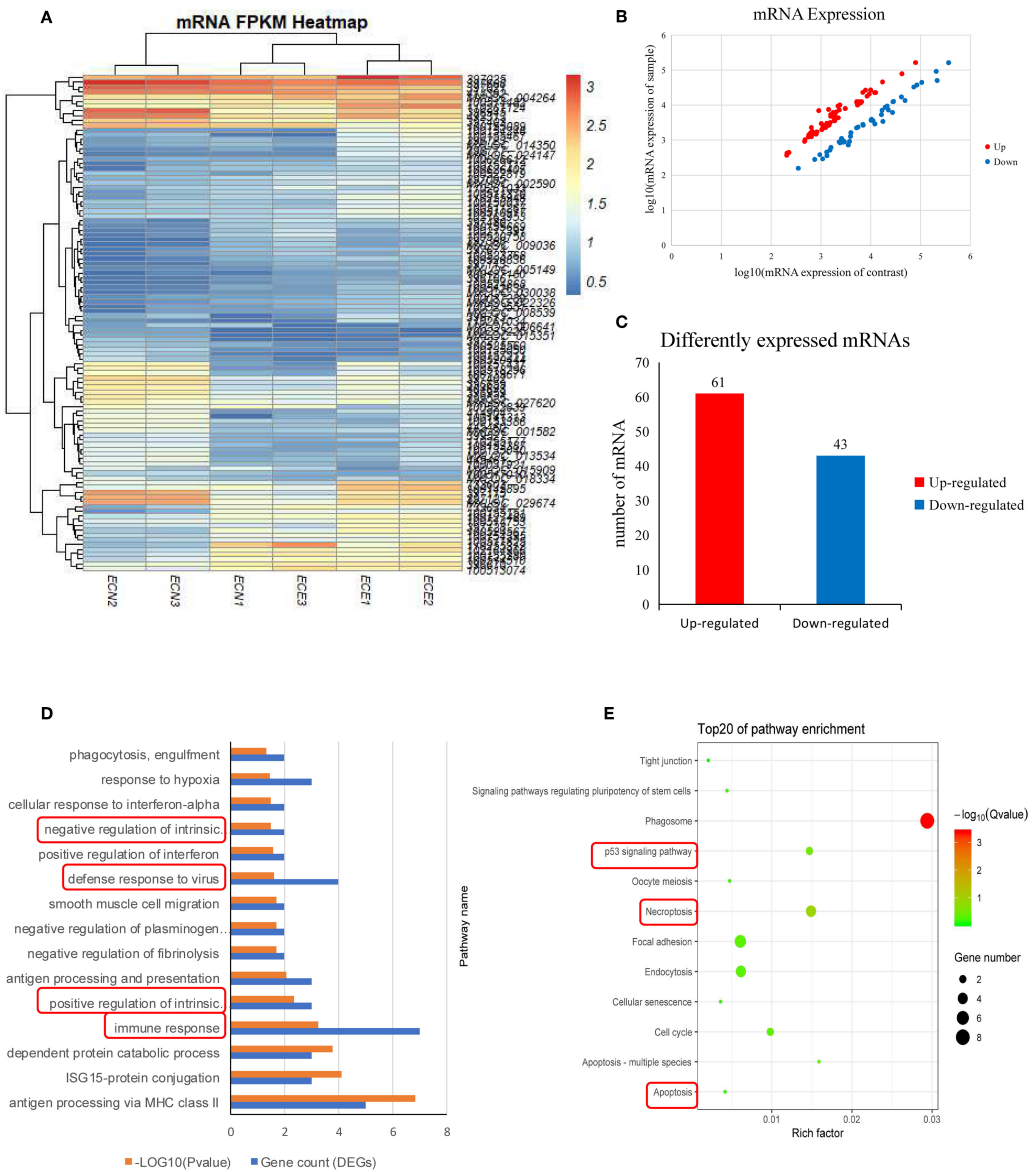
Screening and enrichment analysis of differently expressed genes (DEGs) in PRRSV-infected PECs compared with non-infected PECs. **(A)** Hierarchical clustering analysis (heatmap) for DEGs using Pearson's correlation. **(B)** DEG expression level analysis by scatter plot. The red spots in this figure represent up-regulated mRNAs, the blue spots represent down-regulated mRNAs. **(C)** Detailed analysis on the up-/down-regulated DEGs. Sixty-one up-regulated and 43 down-regulated DEGs were shown. **(D)** The first 20 GO terms analysis (*P* < 0.005) of DEGs in biological process. The red textbox in the figure indicate the main processes that related into cells immunology. A –log_10_ (*P-*value) > 2 was considered as significantly different. **(E)** KEGG signal pathway enrichment analysis of DEGs (*P* < 0.01). The pathways labeled with red textbox were important pathways that are related to cells apoptosis.

Six mRNA expression profiles of PECs were generated from Large White pigs after filtering for reads with low quality and removing adaptor sequences (Table 3), three of each from mock- and PRRSV-infected PECs, respectively. Clean reads were then assembled and 104 DEGs were detected (FC > 2 and FDR < 0.01) (Supplemental Table 4). A heatmap of the DEGs was then constructed and is shown in Figure 3A. mRNA expression levels are displayed in a scatter plot in Figure 3B. Detailed analysis of the up-/down-regulated DEGs is shown in Figure 3C. The identified up-regulated mRNAs may play important roles in PEC damage caused by PRRSV.

**Table 3:** mRNA and lncRNA sequence counts between samples.

**Sample**	**Total raw reads**	**Total clean reads**	**Total clean base**	**Clean reads ratio**
ECE1	133380672	126897516	12689751600	95.14%
ECE2	133380286	126455060	12645506000	94.81%
ECE3	133377020	126587268	12658726800	94.91%
ECN1	133380108	126253656	12625365600	94.66%
ECN2	135026492	126770380	12677038000	93.89%
ECN3	135027010	127240652	12724065200	94.23%

### Enrichment of DEGs

DEGs were then assessed by GO and KEGG pathway analysis. Immune response (*P* = 0.000593), defense response to virus (*P* = 0.0232515), and intrinsic apoptotic signaling pathways (positive, *P* = 0.004421637; negative, *P* = 0.03125) were revealed in the GO analysis (Figure 3D; Supplemental Table 5). Three potential pathways were predicted (p53 signal pathway, necroptosis, and apoptosis; *P* < 0.05), and were mainly involved in cell survival (Figure 3E).

### Summary of lncRNA Sequencing in PECs

**Figure 4:**
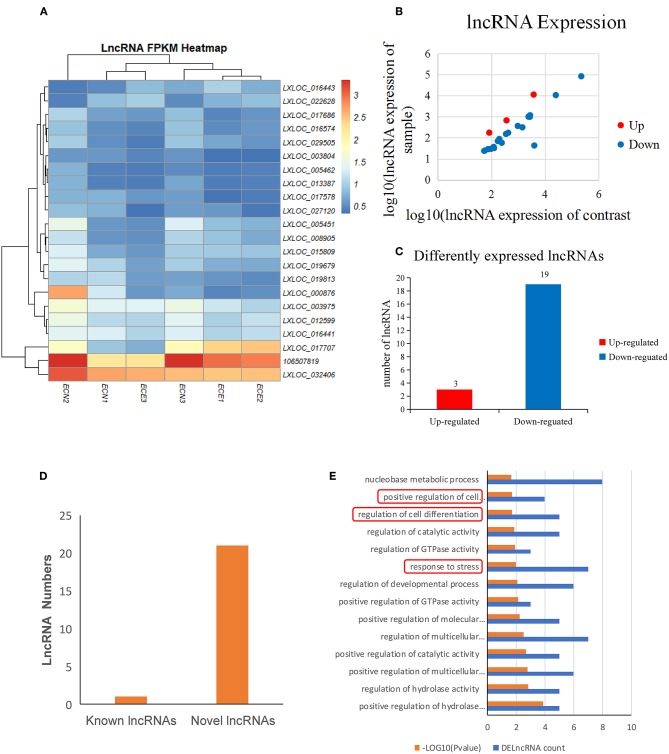
Screening and enrichment analysis of differently expressed lncRNAs (DElncRNAs) in PRRSV-infected PECs compared with non-infected PECs. **(A)** Hierarchical clustering analysis (heatmap) for DElncRNAs using Pearson's correlation. **(B)** DElncRNA expression level analysis by scatter plot. The red spots in this figure indicate up-regulated lncRNAs, the blue spots indicate down-regulated lncRNAs. **(C)** Detailed analysis on the up-/down-regulated DEGs. Three up-regulated DelncRNAs and 19 down-regulated DelncRNAs were shown. **(D)** Known and novel lncRNAs identified in this study. One known DElncRNAs and 23 novel DElncRNAs were shown. **(E)** The first 20 GO terms analysis (*P* < 0.005) of DElncRNAs in biological processes. The red textbox in the figure indicate the main processes that are related into cells immunology and differentiation. A–log_10_ (*P-*value) > 2 was considered as significantly different.

The expression of lncRNAs was then evaluated and a heatmap of the DElncRNAs was created based on the expression levels (Figure 4A; Table 4). A total of 22 DElncRNA genes and 24 potential transcripts were predicted and exhibited obvious changes in the scatter plot (*P* < 0.05) (Figure 4B; Supplemental Table 6). Detailed analysis on the up-/down-regulated DElncRNAs suggested that the down-regulated lncRNAs may play important roles in PRRSV infection processing (Figure 4C). The analysis also revealed that the known lncRNAs represent only a small portion of all DElncRNAs (Figure 4D).

**Table 4:** Differentially expressed lncRNAs.

**GeneID**	**Length**	**Contrast readnum**	**Sample readnum**	**log2Ratio**	**Up/down regulation**	***P* value**	***Q* value**
LXLOC_000876	237	196	88	−1.017573944	Down	1.07E-08	8.45E-09
LXLOC_005451	243	55.88	24.86	−1.030801443	Down	0.002027419	0.000811493
LXLOC_019679	251	54	24	−1.03222072	Down	0.002387418	0.000935437
LXLOC_019813	229	349.04	153.07	−1.051496539	Down	4.51E-15	5.10E-15
LXLOC_017686	2165.79	2768.54	1187.19	−1.08387024	Down	2.08E-113	1.37E-112
LXLOC_027120	352	25173	10710.43	−1.095156477	Down	0	0
LXLOC_016574	599	2415.04	1012.51	−1.116406646	Down	6.04E-104	3.63E-103
106507819	243	421.94	174.17	−1.138837433	Down	3.73E-20	5.14E-20
LXLOC_029505	460	173.29	70.22	−1.165530221	Down	1.93E-09	1.60E-09
LXLOC_017578	234	74.95	29.08	−1.228196927	Down	3.86E-05	2.09E-05
LXLOC_013387	1201	960	368	−1.245624358	Down	3.67E-50	1.10E-49
LXLOC_012599	1114	223984	84141	−1.274810528	Down	0	0
LXLOC_003975	3473	2707	996	−1.304772959	Down	3.16E-148	2.70E-147
LXLOC_032406	252	96	30	−1.540367624	Down	3.13E-08	2.38E-08
LXLOC_008905	238	121	37	−1.57170559	Down	2.88E-10	2.53E-10
LXLOC_016441	215	124.84	32.43	−1.806975052	Down	1.56E-12	1.57E-12
LXLOC_003804	234	242.7	58.27	−1.920644611	Down	9.47E-25	1.52E-24
LXLOC_005462	674	1428	316	−2.038295234	Down	5.45E-148	4.66E-147
LXLOC_015809	262	3960.58	43.36	−6.37549887	Down	0	0
LXLOC_022628	621	3760	11364	1.733370451	Up	0	0
LXLOC_016443	336	83	176	1.222096469	Up	4.43E-11	4.10E-11
LXLOC_017707	874	366.61	675.19	1.01875168	Up	7.68E-29	1.40E-28

### Enrichment of DElncRNA Target Genes

DElncRNA isoforms were then evaluated and 109 isoforms were detected (Supplemental Table 7). GO term analysis of the DElncRNAs hinted that they were mainly involved cell differentiation (*P* = 0.018) and response to stress (*P* = 0.01) (Figure 4E; Supplemental Table 8) in PRRSV infected PECs.

### Integrated Analysis of DEGs and DEmiRNA Target Genes

**Figure 5:**
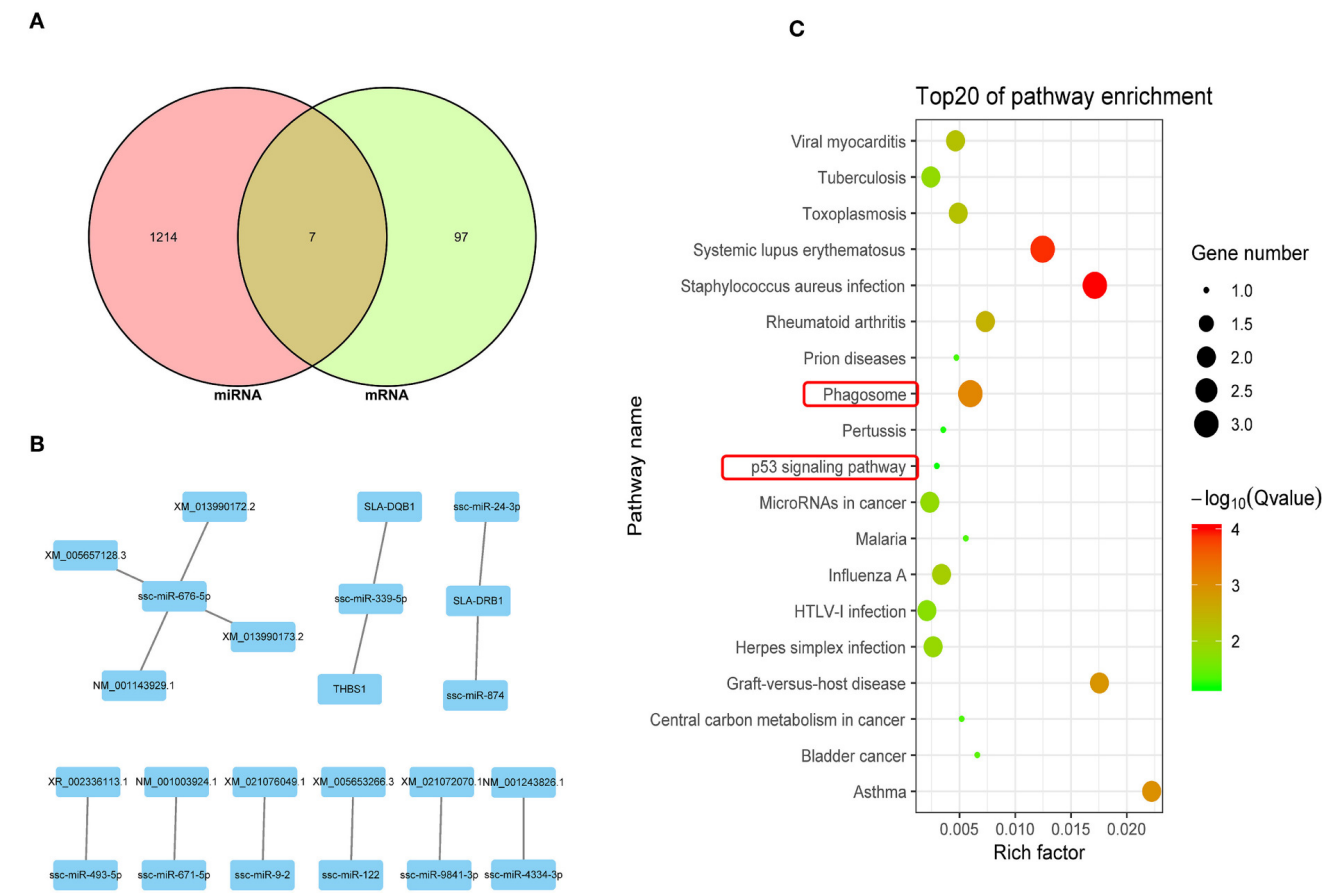
Integrated analysis of DEGs and DEmiRNA target genes. **(A)** Venn map analysis of DEmiRNA target genes and DEGs. Seven co-expressed genes from the DEGs and DEmiRNA target genes were shown. **(B)** Regulatory relationship between DEGs and DEmiRNAs. Nine DEmiRNAs that are related to the DEGs regulatory were shown. **(C)** KEGG analysis based on reciprocal DEmiRNAs and DEGs (*P* < 0.05). The phagosome and p53 signal are the main pathway that are related to cells apoptosis.

An integrated analysis of DEGs and DEmiRNA target genes was carried out to screen for co-expressed genes. The Venn diagram in Figure 5A shows 7 co-expressed genes from the DEGs and DEmiRNA target genes. The relationships between the DEGs and DEmiRNAs were then evaluated and the correlations are shown in Figure 5B (*P* < 0.05) and listed in Table 5. KEGG analysis suggested that the phagosome and p53 signal pathways play important roles during PRRSV infection in PECs (*P* < 0.01) (Figure 5C).

**Table 5:** Differentially expressed miRNAs and corresponding differentially expressed genes.

**miRNA**	**Gene name**	**Fold change**
ssc-miR-339-5p	CD1D	−3.184223813
	SLA-DQB1	−1.549162701
	THBS1	−1.020771929
	SLC3A1	−1.019117703
ssc-miR-324	SLC3A1	−1.019117703
	CAPN3	−3.66965064
	OLFML2B	−4.562735437
	FGL2	−1.704504473
ssc-miR-383	FGL2	−1.704504473
	PALM2	−1.627830465
	HAPLN1	−3.219847723
	ITM2A	−4.385857674
	DRD1	−1.263025381
ssc-miR-874	DRD1	−1.263025381
	SLC2A4	−1.210219022
	CD79A	−4.03222072
	SLA-DRB1	−1.888055067
ssc-miR-24-3p	SLA-DRB1	−1.888055067
	SLC2A4	−1.210219022
	VSIG4	−1.089529403
	MPEG1	−2.658762324
	SIGIRR	−7.057840178
	RLN2	−2.658762324
ssc-miR-181d-5p	ZFP37	−1.199910451
	SELP	−4.562735437
	LOC100517161	−1.325340399

### Integrated Analysis of DEGs and DElncRNA Target Genes

**Figure 6:**
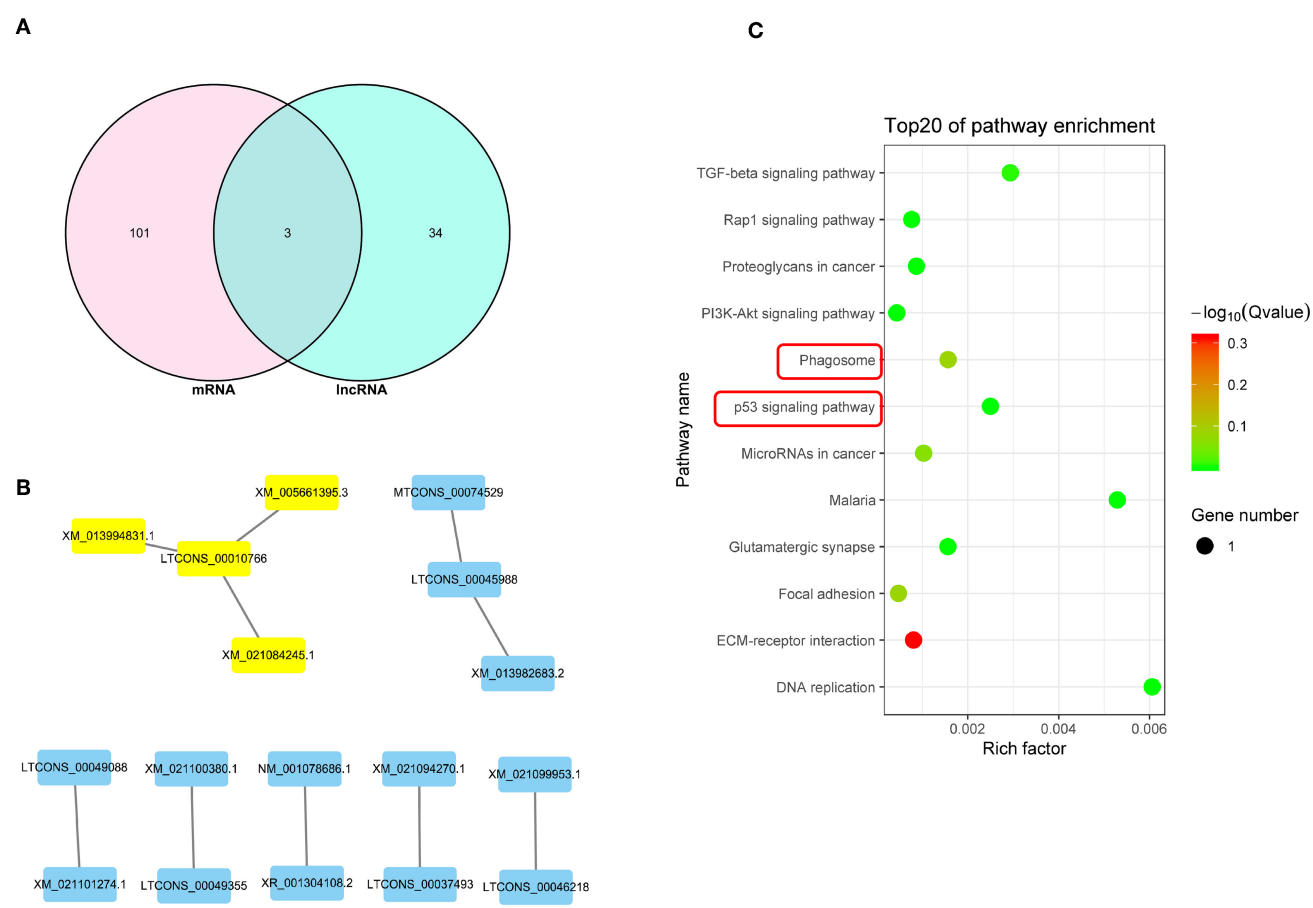
Integrated analysis of DEGs and lncRNAs target genes. **(A)** Venn map analysis between DElncRNA target genes and DEGs.There are 3 co-expressed genes from the DEGs and DElncRNA target genes. **(B)** Regulatory relationship between DEGs and DElncRNAs.There are 8 DElncRNAs that related to the DEGs regulatory. **(C)** KEGG analysis based on reciprocal DElncRNAs and DEGs (*P* < 0.05). The phagosome and p53 signal are the main pathway that related to cells apoptosis.

The Venn diagram shown in Figure 6A illustrates 3 potential genes co-expressed among the DEGs and DElncRNA target genes. DEGs and DElncRNA crosstalk networks were then evaluated and are shown in Figure 6B and listed in Table 6, based on the 10 potential genes regulated by lncRNAs (*P* < 0.05). The phagosome and p53 signal pathways identified in the KEGG pathway analysis suggested that PRRSV could cause cell apoptosis in PECs (*P* < 0.01) (Figure 6C).

**Table 6:** Correlation analysis between differentially expressed mRNAs and lncRNAs.

**GeneID**	**mRNA log2Ratio**	**Annotation**	**Regulation**	***P-*value**	**lncRNA geneID**	**lncRNA log2Ratio**	**Regulation**	***P*-value**	**Pearson_correlation**	**Spearman_correlation**
XM_013994831.1	1.2301	MUM1X7	Up	3.73E-25	LTCONS_00010766	−1.0322	Down	2.39E-03	−0.7028	−0.7714
XM_021084245.1	−7.047	MUM1X12	Down	2.12E-17	LTCONS_00010766	−1.0322	Down	2.39E-03	0.6607	0.6761
XM_005661395.3	5.9011	GAMT	Up	2.77E-08	LTCONS_00010766	−1.0322	Down	2.39E-03	−0.9902	−0.8804
XM_021094270.1	−2.3265	ANKRD27X3	Down	2.37E-72	LTCONS_00037493	−1.1655	Down	1.93E-09	0.8015	0.8857
MTCONS_00074529	−6.1824		Down	1.46E-10	LTCONS_00045988	−1.0515	Down	4.51E-15	0.6741	0.6761
XM_013982683.2	1.9422	FBXO16	Up	2.18E-10	LTCONS_00045988	−1.0515	Down	4.51E-15	−0.7887	−0.6667
XM_021099953.1	−7.0377	RGS6	Down	6.85E-45	LTCONS_00046218	−1.3048	Down	3.16E-148	0.9844	0.8804
XM_021101274.1	−1.8287	ZFP493	Down	3.87E-23	LTCONS_00049088	−1.2282	Down	3.86E-05	0.8703	0.759
XM_021100380.1	−1.3566	ANKRD17	Down	1.03E-95	LTCONS_00049355	−1.5717	Down	2.88E-10	0.6131	0.8286
NM_001078686.1	−1.2773	ODAM	Down	1.93E-03	XR_001304108.2	−1.2748	Down	0.00E+00	0.9484	0.9276

### Genes Related to PRRSV-Induced Apoptosis in PECs

**Figure 7:**
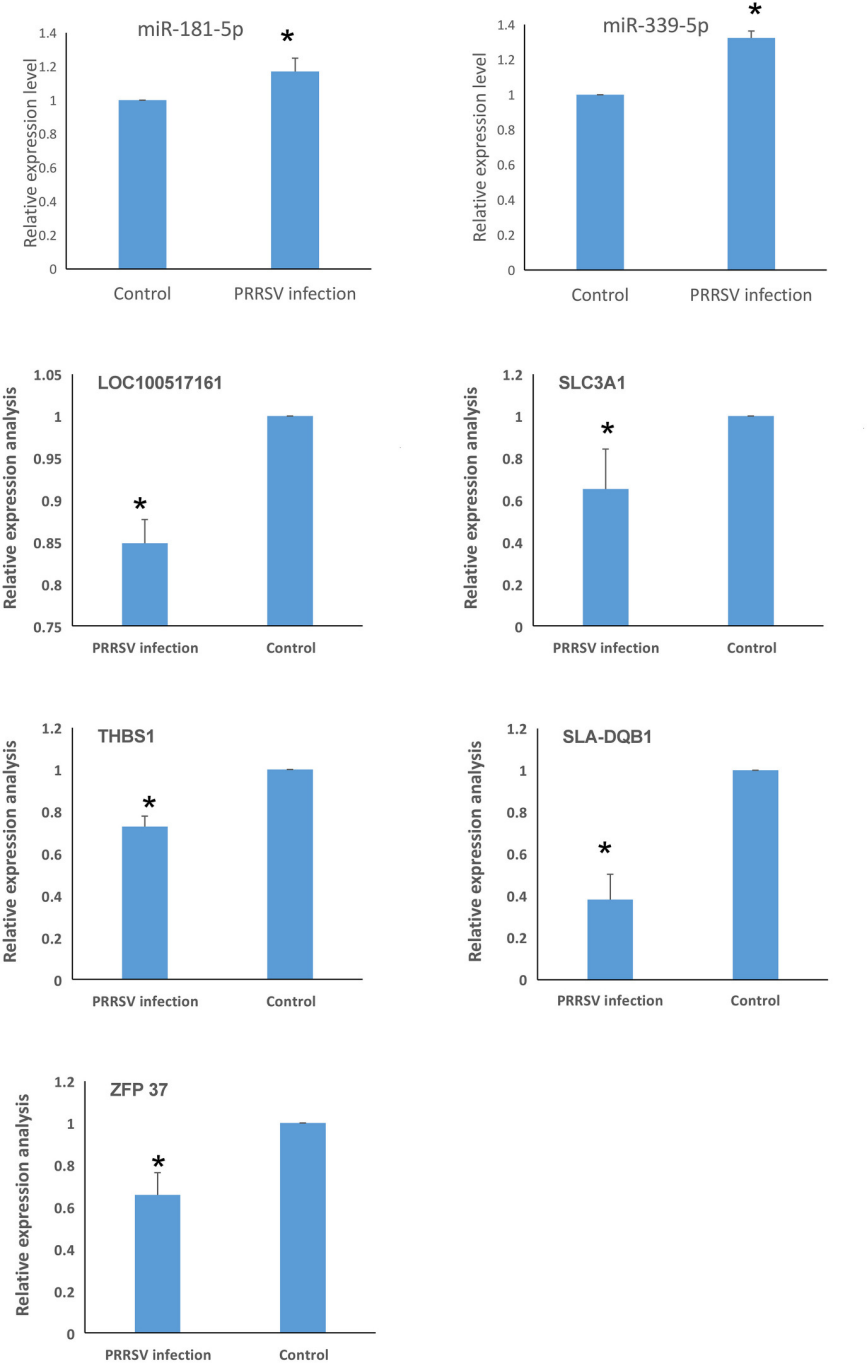
Identification of genes related to PRRSV-induced apoptosis in PECs (^*^P < 0.05). *miR-339-5p* and *miR-181-5p* were significantly up-regulated in PRRSV-infected PECs compared to control cells. The expression of *SLC3A1, THBS1, SLA-DQB1, ZFP37*, and *LOC100517161* were down-regulated in PRRSV-infected PECs compared to non-infected PECs *(P* < 0.05).

Related DEGs and DEmiRNAs that were identified in the crosstalk network analysis were verified by real-time PCR. *miR-339-5p* and *miR-181-5p* were found to be significantly up-regulated in PRRSV-infected PECs compared to the control (Figure 7). Additionally, the target genes of *miR-339-5p* and *miR-181-5p* were evaluated and the expression of *SLC3A1, THBS1, SLA-DQB1, ZFP37*, and *LOC100517161* were found to be down-regulated compared to the control group (Figure 7).

### Functional Analysis of *miR-339-5p*

**Figure 8:**
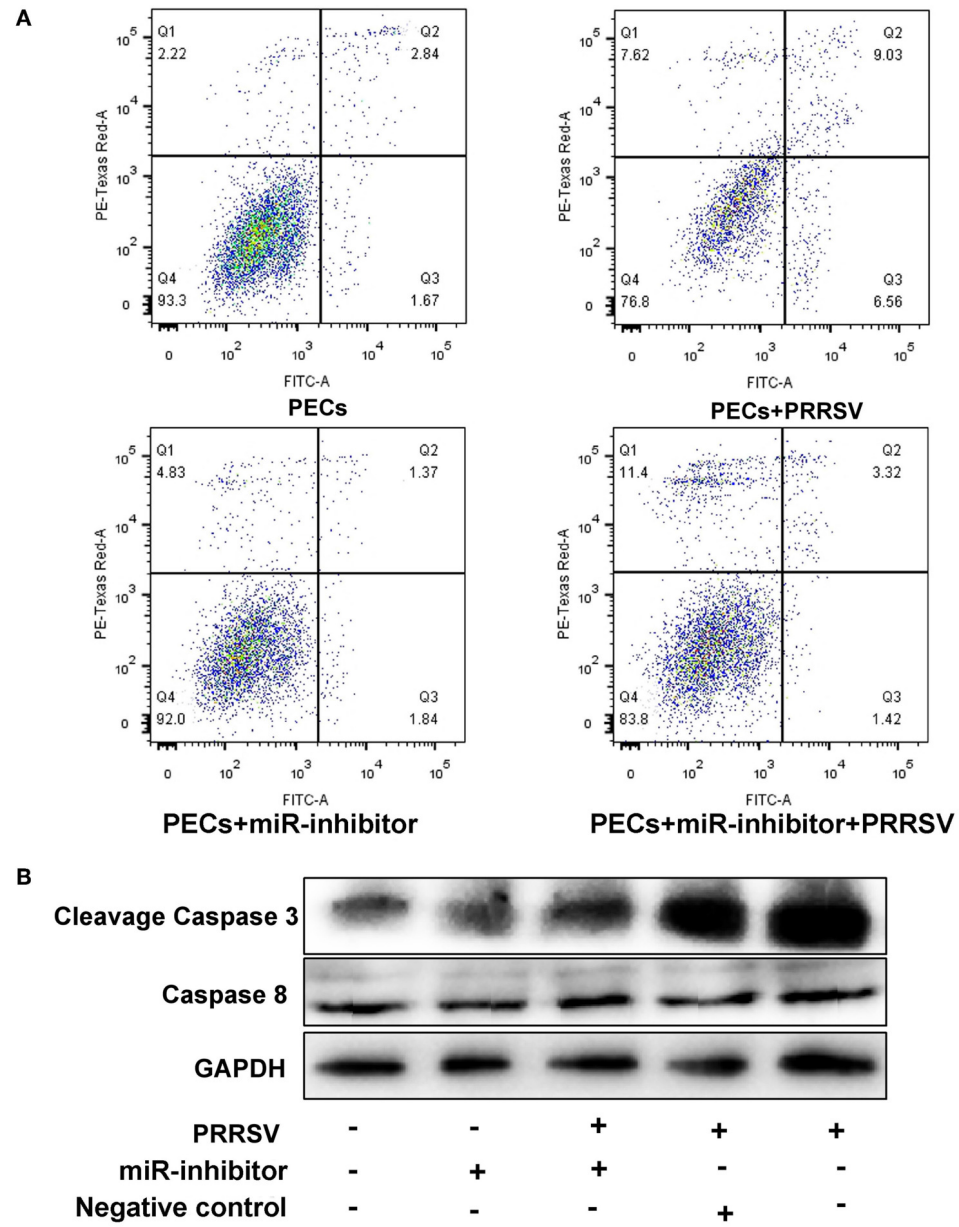
Function analysis of *miR-339-5p* in PECs. **(A)** Apoptosis ratio analysis of PECs with different treatments. The survival ratio of cells transfected with miR-339-5p inhibitor increased compared with the normal PECs upon PRRSV infection. **(B)** Western blotting analysis of Caspase 3 and Caspase 8 protein. The expression of cleavage Caspase 3 protein obviously decreased after transfer miR-339-5p inhibitor, while the expression of Caspase 8 protein showed no obvious changes.

The effect of *miR-339-5p* knock down on the apoptosis of PECs was analyzed. Cells transfected with *miR-339-5p* inhibitor (antisense oligonucleotides) obviously increased its survival ratio compared with the normal PECs upon PRRSV infection (Figure 8A). Western blotting also indicated that *miR-339-5p* could reduce the level of expressed cleavage Caspase 3 rather than Caspase 8 protein (Figure 8B).

## Discussion

In this study, we identified a link between apoptosis of placental cells and PECs and reproductive failure in pregnant sows. We first isolated Sn-positive PECs and examined apoptosis rates by flow cytometry. Apoptosis rates were significantly higher in PRRSV-infected cells than in control cells. Subsequently, the whole PEC transcriptome was analyzed for DEGs and GO/KEGG pathways. Differentially expressed miRNA, mRNA and lncRNA were identified in PRRSV-infected PECs. Integration analysis identified differentially co-expressed target genes and regulatory crosstalk networks. Regulatory crosstalk networks of miRNAs with DEGs and lncRNAs with DEGs were constructed separately. Pathway enrichment revealed that the phagosome and p53 pathways were likely the main signals resulting in cell apoptosis in PECs.

Placental cells and PECs are the main target cells of PRRSV invasion (6). PRRSV could infect CD163- and Sn-positive macrophages in the late period of pregnancy, ultimately resulting in PEC apoptosis (6). Feng et al. isolated and generated a PEC line susceptible to PRRSV (4). The isolated cells in this study expressed CK-18 and Sn protein, which verified the cell type as previously reported (4). A cell apoptosis test confirmed the susceptibility of PECs and verified that PEC apoptosis was caused by PRRSV, as in previous reports (3, 31, 32).

Several studies have focused on miRNA profile variation in PRRSV-infected target cells. Xu et al. analyzed PRRSV-infected PAM cells at different infection time points and screened clusters of miRNAs (33). Li et al. compared miRNA profiles in different pig breeds and obtained 6 DEmiRNAs that may contribute to Landrace-specific responses of PRRSV infection (34). miRNA profile changes were also investigated by Zhou et al. in Marc-145 cells (35). Significant differences exist between our study and others. One difference is that our study mainly focused on the reproductive system (PECs), while others have primarily concentrated on the respiratory system (lung samples, PAM cells, etc.) (32–35). The miRNAs identified in this study are different from those in previous reports. Another difference is that the DEmiRNA enrichment analysis showed enrichment for cell growth, autophagy, and phagosome pathways, which differs from previous reports (35).

mRNA profiles were also investigated in many previous studies, and several tissues and cells were found to be prone to damage caused by PRRSV, such as peripheral blood mononuclear cells (15), lung dendritic cells (36), Marc-145 cells (37), and lung tissues (34). Discrepancies among the differentially expressed mRNAs identified in previous studies may be due to differences in pig breeds and PRRSV strain. In this study, mRNA transcription profiles were created and the data identified 104 DEGs that were mainly enriched in cell apoptosis and the p53 signal pathway. The signal pathways identified in this study are the same as reported previously (5).

The only published research related to lncRNA profile changes was carried out by Zhang et al. in PAM cells (12). They identified 299 DElncRNAs, which were mainly enriched in viral infection and immune response. Their data also suggested that lncRNAs might play regulatory roles in virus–host interactions. Significant differences were found in our study compared with Zhang et al. in PAM cells (12). PAM cells are mononuclear cells sensitive to PRRSV, but the cells used in this study were epithelial cells, which mainly play defensive roles during viral infection. Moreover, Zhang's study mainly focused on the immune system (12), which is different from this study.

Integrated analysis of DEmiRNAs and DEG profiles was carried out to find common genes with altered expression. There were 6 DEmiRNAs and 7 DEGs found in this study. A regulatory network was built based on the correlation between these DEmiRNAs and DEGs. The identified miRNAs (*ssc-miR-339-5p* and *ssc-miR-181d-5p*) and genes (*SLA-DQB1, THBS1, SLC3A1, ZFP37*, and *LOC100517161*) were screened after analysis of genes related to the phagosome and p53 signal pathways. Previous studies reported up-regulation of *miR-339-5p* could activate the p53 apoptosis pathway *via* targeting *MDM2* mRNA in tumor cells; in contrast, down-regulated *miR-339-5p* increased cell proliferation (38–41). *miR-181* has been widely investigated and is known to be a key gene influencing gene expression and cell apoptosis, especially in PRRSV-infected cells (42–46). *miR-181d* acts as a tumor suppressor by targeting *K-ras* and *Bcl-2* (47). *SLA-DQB1* has been recognized as an antigen presentation gene (48, 49). *THBS1* participates in the p53 signal pathway and was found to influence the survival of tumor cells in a previous study (50). Expression of *SLC3A1* enhanced tumorigenesis in tumor cells, whereas inhibition of *SLC3A1* suppressed tumor growth (51).

Analysis of DEG and DElncRNA profiles revealed a correlation between mRNA and lncRNA, and also suggested that the p53 signal pathway is the main pathway that influences PEC apoptosis. The genes that were involved in the p53 signal pathway were then evaluated and compared with previous studies. *LTCONS_00010766* and *LTCONS_00045988* have never been reported in previous studies, but the target genes of these lncRNAs (*MUM1X12, MUM1X7, GAMT*, and *FBXO16*) are known as key genes influencing the p53 pathway (52–56). Functional analysis of *miR-339-5p* exhibited that the *miR-339-5p* gene is involved into p53 pathway, which is as same as previous studies (38–40).

## Conclusions

In summary, we first isolated Sn-positive PECs and then checked apoptosis rates by flow cytometry. Apoptosis rates in PRRSV-infected PECs were significantly higher than in control cells. Whole PEC transcriptome analysis revealed a total of 54 DEmiRNAs, 104 DEGs, 22 DElncRNAs, and 109 isoforms that are mainly involved in apoptosis, necroptosis, and the p53 signal pathway. Integration analysis of DEmiRNA and DEG profiles identified two lncRNAs and five genes that may participate in apoptosis. Integration analysis of DEGs and DElncRNAs profiles showed that target genes by *LTCONS_00010766* and *LTCONS_00045988* were related to the apoptosis signal pathway. Pathway enrichment revealed that the phagosome and the p53 pathways are likely the main signals contributing to cell apoptosis in PECs.

## Ethics Statement

All sows used in this study were housed in livestock housing and fed *ad libitum*. The sacrifice of sows was carried out with sodium barbital after anesthesia. All procedures involving animals were approved by the Animal Care and Use Committee of Shandong Agricultural University.

## Author Contributions

FS, LG, and YJ designed the experiments and drafted the manuscript. KZ, SD, and YL carried out animal care, prepared samples, and performed the experiments. DW and CM performed the data processing and biological information analysis. FS, YW, CZ, and YJ conceived the study and the experimental design, and helped draft the manuscript. All authors have read and approved the final manuscript.

### Conflict of Interest Statement

The authors declare that the research was conducted in the absence of any commercial or financial relationships that could be construed as a potential conflict of interest.

## Abbreviations

DEG: differentially expressed gene
DElncRNA: differentially expressed lncRNA
DEmiRNA: differentially expressed miRNA
FC: fold change
FDR: false discovery rate
GO: gene ontology
miRNA: microRNA
PAM: pulmonary alveolar macrophages
PEC: pig endometrial epithelial cell
PRRSV: porcine reproductive and respiratory syndrome virus
ceRNA: competing endogenous RNAs.
